# Genotypic diversity of the Killer Cell Immunoglobulin-like Receptors (KIR) and their HLA class I Ligands in a Saudi population

**DOI:** 10.1590/1678-4685-GMB-2015-0055

**Published:** 2016

**Authors:** Suliman Y. Al Omar, Afrah Alkuriji, Saleh Alwasel, javid Ahmed Dar, Alwaleed Alhammad, Stephen Christmas, Lamjed Mansour

**Affiliations:** 1 Zoology Department, College of Science, King Saud University, Riyad, Saudi Arabia; 2 Central Laboratory College of Science, King Saud University, Riyad, Saudi Arabia; 3 Immunology Unit, Department of Pathology, King Saud University, Riyad, Saudi Arabia; 4 Department of Clinical Infection, Microbiology & Immunology, Institute of Global Health, Faculty of Health & Life Sciences, University of Liverpool, Liverpool, UK; 5 Unité de Recherche de Biologie Intégrative et Ecologie Évolutive et Fonctionnelle des Milieux Aquatiques, Département de Biologie, Faculté des Sciences de Tunis, Université de Tunis El Manar, Tunisia

**Keywords:** KIR diversity, gene polymorphism, molecular evolution, PCR SSP, population viability analysis

## Abstract

Killer Cell Immunoglobulin-like Receptors (KIR) have been used as good markers for the study of genetic predisposition in many diseases and in human genetic population dynamics. In this context, we have investigated the genetic diversity of KIR genes and their main HLA class I ligands in Saudi population and compared the data with other studies of neighboring populations. One hundred and fourteen randomly selected healthy Saudi subjects were genotyped for the presence or absence of 16 KIR genes and their HLA-C1, -C2, -Bw4^Thr80^ and Bw4^Ile80^ groups, using a PCR-SSP technique. The results show the occurrence of the framework genes (3DL2, 3DL3 and 2DL4) and the pseudogenes (2DP1 and 3DP1) at highest frequencies. All inhibitory KIR (*i*KIR) genes appeared at higher frequencies than activating genes (*a*KIR), except for 2DS4 with a frequency of 90.35%. A total of 55 different genotypes were observed appearing at different frequencies, where 12 are considered novel. Two haplotypes were characterized, AA and Bx (BB and AB), which were observed in 24.5% and 75.5% respectively of the studied group. The frequencies of *i*KIR + HLA associations were found to be much higher than *a*KIR + HLA. KIR genes frequencies in the Saudi population are comparable with other Middle Eastern and North African populations.

## Introduction

Natural killer (NK) cells are considered the first line of defense of the innate immune response against both infected and malignantly transformed cells. They act through their cytotoxic activity or by the production of a panel of cytokines. These activities are accomplished by two subpopulations; CD56^dim^ cells, mainly responsible for cytotoxic activity and CD56^bright^ cells, having a role as cytokine producer cells (Caligiuri *et al.*, 1989, Cooper *et al.*, 2001). The killing activity of NK cells is regulated through a range of receptors belonging to different families. One common family of receptors are called killer cell immunoglobulin-like receptors (KIR) expressed by natural killer cells and a subset of T cells, are glycoproteins playing an important role in innate cytotoxic activity. Through their interactions with Human Leukocyte Antigens (HLA)-I and other ligands, they regulate the cytotoxic activity of the NK cells, by balancing the function of these cells between activating and inhibitory signals (Vilches and Parham, 2002, Parham, 2005, Parham and Moffett, 2013). The KIR genes form a cluster on chromosome 19q13.4 within the Leukocyte Receptor Complex (LRC) in a region spanning approximately 1 Mb (Wagtmann *et al.*, 1997, Torkar *et al.*, 1998). These KIR genes are characterized by their allelic and haplotypic polymorphisms which were associated with the resistance*vs.* susceptibility to many diseases, such as infections, cancers and autoimmunity (Kulkarni *et al.*, 2008a, Al Omar*et al.*, 2010, Hou*et al.*, 2010, Jiao*et al.*, 2010, Garcia-Leon *et al.*, 2011, Augusto *et al.*, 2012, Ivarsson *et al.*, 2013, Jarduli *et al.*, 2013, Chavan *et al.*, 2014).

The KIR gene cluster is composed of 16 genes coding for activating or inhibitory receptors and two pseudogenes (Marsh *et al.*, 2003a). KIR gene nomenclature as defined by the World Health Organization subcommittee was based on the structure of the encoded molecules. Thus according to the number of extracellular immunoglobulin domains (D) which may be double (2D) or triple (3D) and the length of the intracytoplasmic tail that would be long (L) or short (S), the KIR genes have been named and classified as inhibitory (with long cytoplasmic tail) or activating genes (having short cytoplasmic tail) (Long *et al.*, 1996). Actually eight genes were reported coding inhibitory molecules named (KIR2DL1, KIR2DL2, KIR2DL3, KIR2DL5A, KIR2DL5B, KIR3DL1, KIR3DL2 and KIR3DL3) and seven genes for the activating molecules (KIR2DS1, KIR2DS2, KIR2DS3, KIR2DS4, KIR2DS5A/B and KIR3DS1). The gene KIR2DL4 appeared to have both functions (Faure and Long, 2002). KIR2DP1 and KIR3DP1 are pseudogenes (Wilson *et al.*, 2000, Marsh *et al.*, 2003b). Genetic variability in KIR between individuals results from allelic diversity for some genes and the composition of haplotypes (presence or absence of some genes) leading to different genotype composition. Currently, more than 500 different genotypes have been described (Gonzalez-Galarza *et al.*, 2014). Four genes KIR2DL4, KIR3DL2, KIR3DL3 and KIR3DP1 considered as 'framework' are present in nearly all individuals, and for the other genotypes different combinations of genes lead to different haplotypes. Despite the number of the generated haplotypes, two distinct groups were defined, termed A and B, based upon gene content. Group B haplotypes were defined by the presence of at least one the following genes: KIR2DS1/2/3/5, KIR3DS1and KIR2DL5. Conversely, in group A haplotypes all these genes are missing (Uhrberg *et al.*, 2002).

It is now accepted that the killing function of NK cells depends on the composition of activating and inhibitory receptors present on the membrane and the interaction with their HLA ligands. Specificity towards HLA-A, -B, -C and -G ligands has been demonstrated for some KIR molecules (Brooks*et al.*, 2000, Moretta *et al.*, 2014). For HLA-C, based on the amino acid at position 80 in the heavy chain, two groups of ligands were reported. In the HLA-C1 group, position 80 is occupied by the amino acid lysine, while in the HLA-C2 group the amino acid at the same position is asparagine (Moretta *et al.*, 1995). The discrimination between the two groups of HLA-C is allowed by position 44 in the D1 domain of KIR. The inhibitory KIR2DL2/3 and the activating KIR2DS2 bind to the HLA-C1 ligand while KIR2DL1 and KIR2DS1 bind to HLA-C2 (Du *et al.*, 2007). The inhibitory signal triggered by the KIR2DL2/3 + HLA-C1 interaction is relatively weaker as compared with that triggered by the KIR2DL1 + HLA-C2 interaction (Mandelboim *et al.*, 1996, Moesta *et al.*, 2008). The ligand of the receptors KIR3DL1/S1 is the HLA-B allotypes and certain HLA-A molecules that express the Bw4 epitope, serologically defined having a determined motif at amino acid position 77-83 (Cella *et al.*, 1994, Gumperz *et al.*, 1995). According to the amino acid at position 80 of HLA-B having the Bw4 epitope, a dimorphic molecule is expressed; Bw4 Isoleucine and Bw4 Threonine, which affects the strength of interaction with the KIR3DL1 receptor. The inhibitory signal triggered with HLA-B Bw4^Ile80^ is generally stronger than the one triggered with HLA-B Bw4 ^r80^ (Gumperz *et al.,* 1997, Carr *et al.,* 2005). Other KIR-HLA interactions were reported for KIR3DL2 which binds HLA-A3 and HLA-A11, while the association KIR2DL4 -HLA-G remains controversial (Long and Rajagopalan, 2000,Hansasuta *et al.,* 2004,Rajagopalan and Long, 2005, O'Connor *et al.,* 2007).

The activating receptor KIR2DS1 has been shown to bind weakly to HLA-C2 while*KIR2DS2* binds weakly to HLA-C1 (Stewart *et al.,* 2005, Chewning *et al.,* 2007, Foley *et al.,* 2008).

The aim of this work is to contribute to the assessment of the pattern of genetic diversity in 114 healthy Saudi subjects based on the genetic polymorphism of the KIR genes. Our results were used for comparative analysis with other published data for Saudis and neighboring populations. Moreover, the main HLA class I ligands were also typed in order to evaluate the killing function efficiency of the NK cells.

## Materials and Methods

### Study group

Blood samples were obtained from 114 unrelated healthy individuals selected randomly from the Saudi population visiting the King Khaled University Hospital (KKUH), Riyadh, Kingdom of Saudi Arabia. All participants were asked for their consent according to the permit issued by the Ethics Committee of King Saud University for this study. Among this healthy group, 65 were women and 49 men. Genomic DNA was prepared using the DNeasy Blood & Tissue Kit (Qiagen, Valencia, CA, USA).

### KIR and HLA ligands genotyping

KIR genotyping was performed by PCR-SSP for the presence or absence of the 14 KIR genes, *(KIR2DL1, 2DL2, 2DL3, 2DL4, 2DL5, 2DS1, 2DS2, 2DS3, 2DS4, 2DS5, 3DL1, 3DL2, 3DL3, 3DS1*) and the two pseudogenes*(3DP1* and *2DP1).* Genotyping was firstly performed by the commercially KIR typing kit (Miltenyi Biotec, Inc, Germany) according to the manufacturer's recommendations and the results were confirmed by an alternative protocol using a set of primers previously reported by Vilches *et al* (2007).

For HLA-C1, HLA-C2 and HLA-B Bw4 group typing, the same primers reported by Tajik *et al* (2010) were used. For each assay, PCR was performed in a 20 μL final volume containing 4 μL of 5x FIREPol® Master Mix ready-to-use (Solis Biodyne, Estonia), 0.2 pmol of each primer, 50-100 ng of DNA and ultra-pure water (MilliQ). In addition, for each amplification reaction an internal control of the growth hormone gene was amplified using the primers hGH forward (5'-GCCTTCCCAACCATTCCCT TA-3') and hGH reverse, (5'-GTCCATGTCCTTCCTGA AGCA-3') (Tajik*et al.*, 2009, 2010). The PCR cycling protocol used consisted of an initial step of denaturation at 94°C followed by 5 cycles of 20 s at 94°C, 30 s at 64°C and 60 s at 72°C, 25 cycles of 94°C for 30 s, 60°C for 30 s, and 72°C for 90 s; 5 cycles of 94C° for 30 s, 55°C for 30 min, and 72°C for 90s, followed by a final extension step for 10 min at 72°C. All PCR assays were performed with the Techne TC-Plus Satellites (Bibby Scientific, Staffordshire, UK) thermocycler apparatus.

The PCR products were analyzed by electrophoresis in 2% agarose gel stained with ethidium bromide and visualized on an UV transilluminator using a gel documentation system (Gel225 DocXR BioRad) to check the presence or absence of gene-specific amplicons.

### Statistical analysis

The frequencies of the KIR genes and KIR AA and BX genotypes, as well as their ligands HLA-C1 and -C2 groups and HLA-B Bw4 were established by direct counting. The significance of differences of KIR gene proportions between our sampled populations and other selected ones were estimated using the two-tailed Fisher's exact test with SigmaPlot software version 11. P values of < 0.05 were considered to confirm significance of differences. All values in the studied groups were in Hardy–Weinberg equilibrium. Principal component analysis (PCA) and estimation of the Euclidean distances were performed with Primer E-6 Package(Clarke, 1993) to minimize the genetic distances between our population and other selected ones.

## Results and Discussion

**Figure 1:**
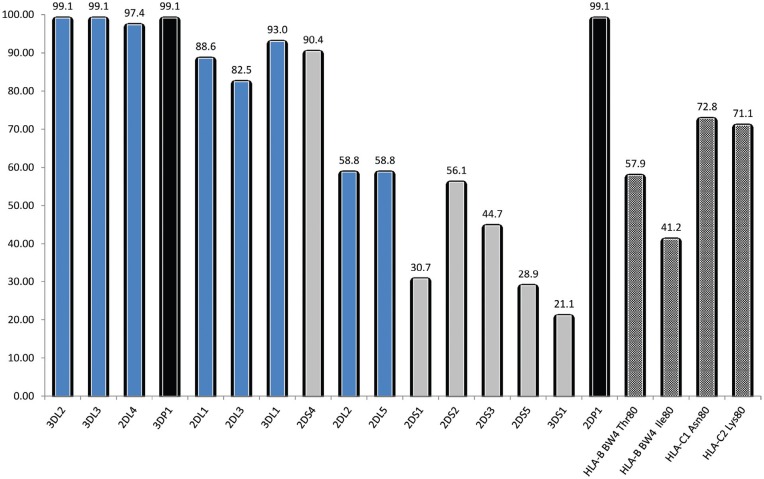
Distribution of observed frequencies of KIR genes and their HLA ligands among the studied Saudi population. Blue = inhibitory, grey = activating, black = pseudogenes, hatched = HLA ligands.

The aim of this work was to assess the KIR gene diversity in a Saudi group of 114 randomly selected healthy unrelated individuals. For this purpose, we used two protocols based on the Sequence-Specific Primer–directed Polymerase Chain Reaction (SSP-PCR). All samples were typed with a commercially KIR typing kit (Miltenyi Biotec, Inc) using unknown primers and a protocol based on the primers designed byVilches *et al.* (2007). Both protocols gave 100% concordant results. The distribution frequencies of the 16*KIR* genes and their HLA class I ligands in a randomly selected 114 healthy unrelated Saudi group are reported in Figure 1.

The framework genes *KIR3DL2*, -*DL3* and -*3DP1* appeared in 99.1% (113/114) of the subjects. The framework gene *2DL4* was observed in 97.4% (111/144). The inhibitory genes *2DL1, 2DL3, 3DL1* appeared with relatively high frequencies (> 80%) and only *2DL2* and *2DL5* were observed with the same frequency at 58.8%. The second pseudogene *2DP1* had the same frequency as *3DP1*.

Comparisons of these frequencies with those of 12 other studied populations including two Saudi groups were made (Tables 1 and2). We noticed that while the percentages of the framework genes did not reach 100%, they are in the range of all reported studies (P < 0.05). For both studied Saudi groups, these percentages were 100% for all framework genes (Gaafar *et al.,* 2011, Osman *et al.,* 2014). For these genes, only the percentages in a group from Southern Turkey reported by Ozturk *et al.* (2012) are less than 100%. These framework genes were lacking in three individuals. Two individuals were A haplotypes and lacking two framework genes simultaneously *(3 DL3, 2DL4 and 3DL2, 2DL4).* These individuals would be homozygote for the Kir haplotypes and could carry a large deletion spanning these loci, probably as a consequence of unequal crossing over events. However, errors caused by limitations in the techniques cannot be excluded. Sequencing of these unusual two individuals should confirm this information. Double deletion of the framework genes were also reported in other studies, such as of the admixed population of Belem, in the Northern region of Brazil and in a Swedish study group (Martin *et al.,* 2008,Pedroza *et al.,* 2011,Gonzalez-Galarza *et al.,*2014).

**Table 1:** Comparative analysis of the distribution of KIR genes between different related populations used for comparisons with our studied Saudi population group (Sap3).

A. Observed percentages of KIRs genes reported in different studied groups.														
KIR genes		Sap3	Sap2	Sap1	Pal	Leb	Oma	Ira-Ar	Ira	Tun	Mor-Ch	Sen	Turk	Ind
		(n=114)	(n=148)	(n=162)	(n=105)	(n=120)	(n = 99)	(n = 76)	(n = 200)	(n=114)	(n = 67)	(n= 118)	(n = 200)	(n = 139)
Inhibitory genes	3DL2	99.12	100	100	100	100	100	100	100	100	100	100	99.5	100
	3DL3	99.12	100	100	100	100	100	100	100	NR	97	NR	99.5	100
	2DL4	97.37	100	100	100	100	100	100	100	100	100	100	99	100
	2DL1	88.6	98	96.3	83	99.2	98	100	96.5	99.1	95.5	100	97	97.8
	2DL3	82.46	83.1	91.4	85	88.3	87.9	89.5	86.5	91.2	73.1	100	80	85.8
	3DL1	92.98	94.4	95.7	88	95.8	96	85.5	91.5	100	100	99	91	91.4
	2DL2	58.77	68.2	56.2	62	59.2	50.5	63.1	56.5	59.6	70.1	55	53	62
	2DL5	58.77	64.9	56.2	63	58.3	59.6	67.1	61.5	60.5	67.2	52	58	71.7
Activating genes	2DS1	30.7	43.9	33.3	44	40.8	32.3	42.7	45.5	22.8	25.4	13	38	47.8
	2DS2	56.14	73	57.4	64	59.2	49.5	56.3	57.5	59.6	65.7	42	53	62.3
	2DS3	44.74	43.2	38.3	37	37.5	30.3	50	38	38.6	52.2	24	33	44.2
	2DS4	90.35	92.5	93.8	88	95	94.9	98.7	91.5	96.5	100	100	92	90.7
	2DS5	28.95	43.9	27.8	27	30.8	39.4	35.5	40	23.7	32.8	30	39	46.4
	3DS1	21.05	35.8	34.6	39	35.8	29.3	42.1	44.5	23.7	26.9	4	42	37
pseudogenes	2DP1	99.12	98	86	NR	NR	98	98.7	96.5	NR	100	NR	96	97.8
3DP1	99.12	100	100	NR	NR	100	100	100	100	100	100	99	NR	
References		Present work	(Gaafar *et al.,* 2011)	(Osman *et al.,* 2014)	(Norman *et al.,* 2001)	(Rayes *et al.,* 2008)	(Williams *et al.,* 2004)	(Norman *et al.,* 2001)	(Hiby *et al.,* 2010)	Unpublished	(Hollenbach*et al.,* 2010)	(Denis *et al.,* 2005)	(Ozturk *et al.,* 2012)	(Kulkarni *et al.,* 2008b)

B. Statistical significance of the distribution of frequency of KIR genes of Sap3 *vs.*related populations. Values in Bold indicate the significance of difference (p < 0.05).														
KIR genes			Sap2	Sap1	Pal	Leb	Oma	Ira-Ar	Ira	Tun	Mor-Ch	Sen	Turk	Ind
Inhibitory genes	3DL2		0.91	0.88	0.95	0.99	0.92	0.82	0.77	0.98	0.77	0.99	0.71	0.94
	3DL3		0.91	0.88	0.95	0.99	0.92	0.82	0.77	0.98	0.61	0.99	0.71	0.94
	2DL4		0.17	0.15	0.29	0.24	0.31	0.42	0.09	0.26	0.48	0.25	0.55	0.19
	2DL1		**0.003**	**0.025**	0.34	**0.001**	**0.0013**	**0.005**	**0.012**	**0.002**	0.19	**Inf 0.001**	**0.002**	**0.004**
	2DL3		1	**0.038**	0.76	0.26	0.34	0.25	0.42	0.072	1.18	**Inf 0.001**	0.71	0.59
	3DL1		0.84	0.53	0.22	0.56	0.57	0.13	**0.80**	**0.014**	0.073	**0.043**	0.61	0.73
	2DL2		0.14	0.75	0.74	0.94	0.27	0.65	0.78	099	0.17	0.65	0.37	0.7
	2DL5		0.42	0.68	0.69	0.98	0.92	0.35	072	0.96	0.33	0.29	0.95	**0.046**
Activating genus	2DS1		**0.044**	0.79	0.068	0.15	0.96	0.16	0.014	0.2	0.51	**0.001**	0.26	**0.015**
	2DS2		**0.007**	0.95	0.31	0.75	0.38	0.91	0.9	0.7	0.27	**0.044**	0.65	0.37
	2DS3		0.93	0.35	0.32	0.33	**0.044**	0.55	0.29	0.43	0.41	**0.001**	0.052	0.98
	2DS4		0.75	0.46	0.58	0.30	0.35	0.052	0.89	0.13	**0.02**	**0.002**	0.86	0.85
	2DS5		**0.014**	0.99	0.86	**0.81**	0.12	0.39	0.066	0.48	0.66	0.97	**0.001**	0.32
	3DS1		**0.012**	**0.019**	**0.005**	**0.017**	0.21	**0.003**	**Inf 0.001**	0.74	0.47	**0.0001**	**0.0001**	**0.009**
Pseudogenes	2DP1		0.77	**0.0001**	**NR**	NR	0.87	0.98	0.29	NR	0.77	0.99	0.87	0.86
	3DP1		0.91	0.88	**NR**	NR	0.92	0.82	0.77	0.98	0.77	0.99	**0.65**	NR

Sap1 and Sap2 are two studied groups of Saudi population; Pal, Palestinian; Leb, Lebanese; Oma, Omani; Ira-Ar, Iranians-Arab; Ira, Iranians; Tun, Tunisians; Mor-Ch, Moroccans Chaouya; Sen, Senegalese; Turk, Southern Turkey; Ind, Indians

**Table 2:** Statistical comparisons of AA /Bx genotypes between our population SaP3 and some related populations. Values in bold for p < 0.05.

Populations		Sap3	Sap2	Sap1	Pal	Leb	Oma	Ira-Ar	Ira	Tun	Mor-Ch	Sen	Turk	Ind
Genotypes	AA	24.5	18.2	26.5	22.9	26.7	29.3	15.7	27.5	29.8	16.4	53	29	23.7
%	Bx	75.5	81.8	72.9	77.1	73.3	70.7	83.3	72.5	70.2	83.5	47	71	76
p	AA/Bx	–	0.28	0.76	0.91	0.80	0.51	0.23	0.33	0.43	0.28	**< 0.001**	0.44	0.98

The frequency of *2DL1* here reported is significantly different to almost all studied populations except the Palestinians and the Moroccan Chaouya groups. The highest frequency (100%) of this gene occurs in Senegalese, Iranian-Arab and Indian groups. Thus, only the Palestinian and the Moroccan Chaouya groups have no significant differences in the distribution of frequencies of inhibitory genes. Our Saudi group has only one significant difference in the frequencies of*i*KIR genes with Saudi S2 group, Lebanese, Omani, Iranian-Arab, and Turkish groups and two significant differences with Saudi S1, Tunisian, Iranian and Indian groups. The most divergent was the Senegalese population with three significant differences in *2DL1, 2DL3* and *3DL1*genes.

For the activating genes only the Tunisian group showed no significant differences in the frequencies of *a*KIRs with our studied group. One significant difference was observed with Saudi S1, Palestinians, Lebanese, Omani, Iranians-Arab, Iranian and Moroccan Chaouya. The Saudi S2 has four significant differences with our group. The Senegalese group remains the most divergent with five significant differences. And finally, in the distribution of the pseudo-genes, only the*2DP1* in Saudi S1 appears lower than all the reported populations.

Based on the presence or absence of inhibitory and activating *KIR*gene content, two major haplotype groups A and B were considered. The homozygote AA haplotype is defined by the absence of all the following genes, *KIR2DL2, −2DL5, −2DS1, −2DS2, −2DS3-2DS5* and *-3DS1.* Conversely, individuals carrying at least one of these genes are considered Bx genotype grouping AB and BB haplotypes.

**Figure 2:**
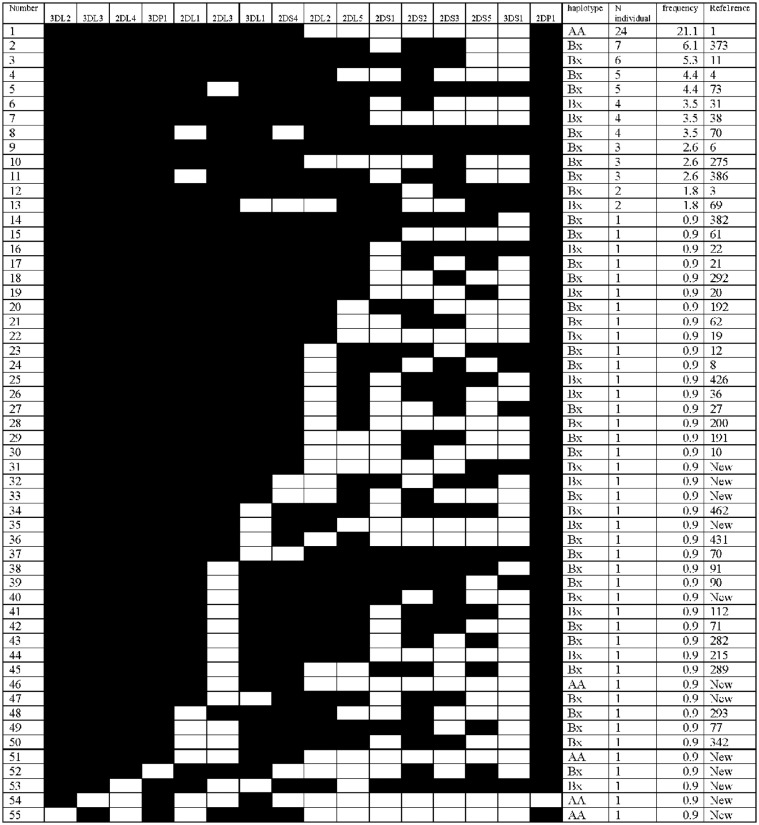
Characterization and distribution of KIR genotypes observed in 114 Saudis individuals. Presence and absence of genes are indicated by filled and open rectangles, respectively.

Identification of *KIR* genes among the studied Saudi group showed the occurrence of 55 different genotypes (Figure 2). These genotypes were characterized by referring to the allele frequencies database (http://www.allelefrequencies.net) (Gonzalez-Galarza *et al.*, 2014). Among the 55 genotypes identified 12 of them were new. Thirteen genotypes have frequencies > 1% and they represent 63% of the studied groups. The homozygote AA referenced as haplotype number 1 is the most frequent with 21.1%. This is the case for the two last reported Saudi groups (Sap1 and Sap2) and most other studied populations. Comparison made with the twelve selected groups, showed a significant difference in the frequency of haplotypes AA and BB only with the Senegalese study group (p < 0.001) (Table 2). In this African population we noted a frequency of the AA haplotype higher than Bx (42% *vs.* 38%). We noted the presence of the reference genotype number 6 with only 2.6%, which was reported at 8.8% in Sap1 and 8.7% in Sap2. The number of genotypes in common with Sap1 and Sap2 were 11 and 12 respectively. We recall that in these two Saudi groups 41 genotypes were reported in the Sap1 and 31 genotypes in the Sap2. Only five genotypes were common to the three subpopulations which are the references 1, 4, 6, 70 and 112. Thus, for a total of 127 genotypes observed in these Saudi groups, only 23 were observed in at least two groups, the remaining 104 genotypes were reported individually. This data suggest a high heterogeneity and diversity in the Saudi population.

Knowing the importance of some HLA ligands in the interactions with the KIR receptors, we have also studied the distribution of the frequencies of HLA-C1 and C2 and HLA Bw4 in this Saudi group (Figure 1). HLA-C1 and -C2 groups appeared with similar frequencies (72.8 and 71.05% respectively). HLA-B Bw4 occurred in 73.7% of the subjects. The groups HLA-B Bw4^*Thr80*^ and HLA-Bw4^*Ile80*^ were observed in 57.9% and 41.2% respectively of the typed individuals.

Table 3 shows the different genotypes of HLA KIR ligands observed in the study population. Six genotypes were observed. The most frequent was Bw4 + C1 + C2 carried by 29.8% individuals. Those carrying two different ligands were more frequent than those with only one ligand (Table 3). These results corroborate with those reported in the Iranian population (Tajik *et al.*, 2010).

**Table 3:** Different associations of HLA KIR ligands among the studied Saudi population

Ligands	C1	C2	B Bw4	number	%
C1/C2/B Bw4	+	+	+	34	29.82
C1/C2	+	+		15	14.03
C1/B Bw4		+	+	27	23.68
C2/B Bw4	+		+	23	20.17
C1	+			6	5.26
C2		+		8	7.01

Analysis of different combinations between KIR genes and their supposed HLA ligands are presented in Table 4. We note that for the *i*KIR, the frequency of the individual sharing*2DL2/3*-C1 appeared with the highest frequency.*2DL1*-C2 and *3DL1*-Bw4 were relatively high and had the same percentage. Conversely, the distribution of *a*KIR-HLA ligand is variable (Table 4). For the HLA-C1 ligand, it was observed associated with *2DL2/3* in 73.68% of the subjects against 42.09% for the *2DS2*. The genotype *2DL2/3, 2DS2*, C1 was observed in 28.9%. The percentage of*2DL2/3* positive individuals without C1 was 27.27%*vs.* 13.15% for *2DS2*.

**Table 4:** Distribution of *i*KIR and *a*KIR and their HLA ligands

	KIR + HLA	% of individuals
iKIR + HLA	2DL2/3 + C1	73.68
	2DL1 + C2	66.67
	3DL1+ Bw4	66.67
	3DL1 +B Bw4 Ile80	37.71
	3DL1 +B Bw4 Thr80	50.87
aKIR + HLA	2DS2 + C1	42.09
	2DS1 + C2	20.17
	3DS1 + Bw4	19.2
	3DS1 +B Bw4 Ile80	8.8
	3DS1 +B Bw4 Thr80	17.5

The ligand HLA-C2 had a frequency of 71%. Its association with*KIR2DL1* gave a stronger signal than the association*2DL2/3*-C1. The association *2DL1*-C2 was observed with a frequency of 66.67% *vs.* 20.17% for the association of this ligand with the activating KIR *2DS1*. The genotype*2DL1-2DS1* was observed in 24.7% of individuals. Their simultaneous association with C2 ligand was observed in 18.4% cases only. 21.9% of the *2DL1*+ and 34.3% of the *2DS1*+ groups were C2 free.

HLA-B Bw4 occurred in 73.68% of the study group. 66.6% are *3DL1*-Bw4+*vs.* 19.2.3% for the genotype *3DS1*-Bw4. The genotype *3DL1, 3DS1*, Bw4 was observed in 16.6% of cases. We have observed 27.3% of *3DL1* and 4.6% of *3DS1*individuals without the B Bw4 ligand.

**Figure 3:**
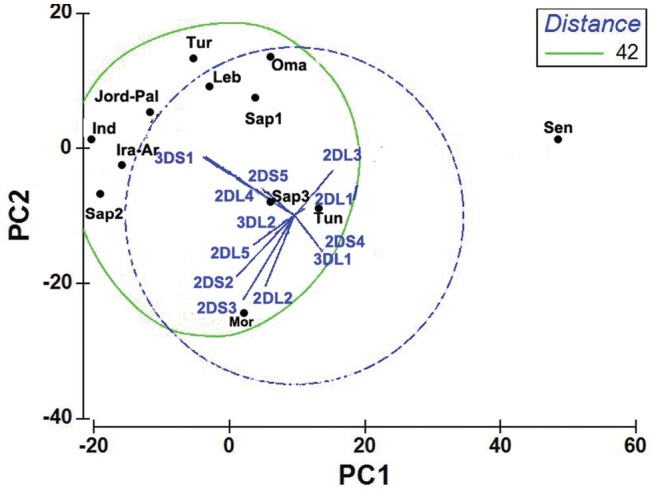
Spatial distribution along the first and second principal components (PC1 and PC2) grouping 74.2 of the variance of 12 KIR genes frequencies from our studied Saudi population (Sap3) and 11 other previously described populations). The relative contribution of each KIR gene to variability is represented by blue arrows. Circles indicate two different clusters obtained for two Euclidean distances (21 for the circle in blue and 42 for the circle in green). For an Euclidean distance 42, only the Senegalese population is out of the cluster.

A principal component analysis mapping of the 12 selected populations based on the frequencies of 12 *KIR* genes is represented in Figure 3. The results show that our Saudi population Sap3 clusters well with other Asiatic and North African populations. The Senegalese population appears quite divergent and is located away from all other groups. It is worth mentioning that Sap3 is genetically closely related to the Tunisian population. The Sap1 group occurs in a same cluster with the Turkish, Lebanese and Omani population. However, the Iranians-Arab population shows some genetic distance from the Sap2 sample.

These results corroborate with other paleoanthropic and genetic studies showing the common origin of the Saudis and other population belonging either to the Arabian Peninsula, Levant, African populations and other Asiatic ethnics groups (Abu-Amero *et al.*, 2007, Abu-Amero *et al.*, 2008, Cadenas *et al.*, 2008, Abu-Amero *et al.*, 2009, Abu-Amero *et al.*, 2011, Rosa and Brehem, 2011, Soares *et al.*, 2012, Abu-Amero *et al.*, 2013, Alsmadi *et al.*, 2013). In fact, according to the 'out of Africa theory' model, Saudi Arabia is thought to have played a key role in the dispersion of early human populations outside of Africa, mainly through the Sinai Peninsula and Bab-el-Mandeb routes probably since the last glacial period. Later, these people continued to spread to the Near East through the Levantine corridor (Petraglia, 2003, Alsmadi *et al.*, 2013).

Through the KIR data analysis we confirmed that the Saudi Arabia population has mainly been a recipient of gene flow from the surrounding areas in Asia and Africa. Explorations *KIR* diversity of other admixed and unique populations of the Arabian Peninsula is very interesting and can provide more information about the evolutionary history and the dynamic of this ethnic group.
